# The effect of bacteria, enzymes and inulin on fermentation and aerobic stability of corn silage

**Published:** 2012-12

**Authors:** S Peymanfar, RK Kermanshahi

**Affiliations:** Department of Biological Sciences, Alzahra University, Vanak, Tehran, Iran

**Keywords:** silage, LAB, microencapsulation, inulin, Enzyme

## Abstract

**Background and Objectives:**

Ensiling is a conservation method for forage crops. It is based on the fact that anaerobe lactic acid bacteria (LAB) convert watersoluble carbohydrates into organic acids. Therefore, pH decreases and the forage is preserved. The aim of this study was to isolate special kinds of lactic acid bacteria from silage and to study the effect of bacteria, inulin and enzymes as silage additives on the fermentation and aerobic stability of the silage.

**Materials and Methods:**

The heterofermentative LAB were isolated from corn silages in Broujerd, Iran and biochemically characterized. Acid tolerance was studied by exposure to acidic PBS and growth in bile salt was measured by the spectrophotometric method.

**Results:**

The results of molecular analysis using 16SrDNA sequences showed that the isolates belonged to Lactobacillus and Enterococcus genera. To enhance stability in acidic environment and against bile salts, microencapsulation with Alginate and Chitosan was used. The Lactobacillus plantarum strains were used as control. The inoculants (1 × 10^7^ cfu/g) alone or in combination with inulin or in combination with enzymes were added to chopped forages and ensiled in 1.5-L anaerobic jars.

**Conclusion:**

Combination of the isolates Lactobacillus and Enterococcus with inulin and enzymes can improve the aerobic stability of corn silage.

## INTRODUCTION

In order to improve the ensiling process, various chemical and biological additives have been developed. The biological additives are advantageous because they are safe and easy to use, non corrosive to machinery, do not pollute the environment, and are regarded as natural products (1). Bacterial inoculants are added to silage in order to stimulate LA fermentation, accelerating the decrease in pH, and thus improving silage preservation. Most available inoculants consist of selected strains of homofermentative LAB, such as *Lactobacillus plantarum*, Pediococcus, and *Enterococcus* species (2, 3). Many studies have shown advantages of such LAB inoculants indicating that addition of homofermantative LAB inoculants improved the aerobic stability of silages of mature cereal crops (wheat, sorghum, maize) (4, 5). This was suggested by rise in pH, visible mould growth, and intensive production of CO_2_ during aerobic exposure.

For many years, investigators have used microencapsulation technique to increase stability and survival of probiotics. Nucleotide base sequences of *Lactobacillus* 16S ribosomal DNA (rDNA) provide an accurate basis for phylogenetic analysis and identification (6). Microencapsulation of the probiotic cells is one of the most recent and efficient methods by which bacteria is prevented from detrimental factors of environments such as: high acidity (low pH), bile salts presence of molecular oxygen in case of obligatory anaerobic microbes, bacteriophages and chemical antimicrobial agents. The encapsulation of viable bacteria cells is carried out by alginate which is a linear heteropolysaccharide extracted from different types of algae, with two structural units consisting of D-mannuronic and L-guluronic acids. Calcium alginate has been widely used for the encapsulation of lactic acid and probiotic bacteria. Chitosan is another material for encapsulation and is a linear polysaccharide with a negative charge arising from its amine groups produced by the deacetylation of chitin. Chitosan has been used for coating of alginate capsules (7–10). Earlier observations had resulted in the opposite that LAB inoculants improved aerobic stability of silages.

The aim of this study was to isolate special kinds of probiotics from silage and to stabilize it for survival in harsh environment and to study the effect of bacteria + enzyme + inulin mixture as silage additives on the fermentation characteristics and aerobic stabilities of corn silage. The addition of enzyme preparations either alone or combined with inoculants was proposed as a strategy to increase available substrate to improve lactic acid fermentation in silage and/or to increase nutritive value of the forage.

## MATERIALS AND METHODS

### Isolation and identification

Silage was added into saline solution (0.85%, pH 7) and shaken by hand. Then serial dilution was prepared and suspension were added into the tubes contain of 10 l MRS broth (Merck) for enrichment. Then tubes were incubated in an anaerobic jar for *Lactobacillus* and aerobically for *Enterococcus* at 37°C (11–13). After one day incubation, tubes that contain turbidity were spread on MRS agar Petri dishes. After 2 days incubation at 37°C, pure culture achieved. LAB was detected by the presence of yellowish colony. One colony was selected for identification. The biochemical standard tests, including Gram staining, catalase reaction, carbohydrates fermentation (Glucose, Galactose, Lactose, Manitol, Fructose, Arabinose, Sucrose, Ribose for *Lactobacillus*), (Glucose, surbose, sucrose, Ribose, arabinose, Manitol, Galactose for *Enterococcus*), growth at 10°C and 50°C in MRS broth and arginine hydrolysis were performed (11–13). For test of carbohydrates fermentation, base MRS broth without glucose and meat extract was used. It contained 0.5% different sugars and 0.004% purple bromo creosl. For arginine hydrolysis test, base MRS broth without glucose and meat extract was used, but it contained 0.3% arginine and 0.2% sodium citrate instead of ammonium citrate (11–13).

### Molecular identification of the selected isolates using 16 SrDNA sequencing

Genomic DNA was extracted from the bacteria with SET buffer method Signoretto (14). Partial sequencing of 16S rDNA of two selected isolates was carried out using to universal primers: forward primer P259 5 > -CCTACGGGAGGCAGCAG –3> and reverse primer Y119 5- GACGTCRTCCNCDCCTTCCT-3. The PCR reactions and cycles were performed according to the method of Lleo‘, *et al*. (14).

The PCR amplification was carried out using a GeneAmp PCR 2700 system (Applied Biosystems, Foster City, CA), using an initial denaturation step of 3 min at 95°C, followed by 1 cycle of denaturation at 95°C for 2 min, 30 cycles annealing at 55°C for 1 min, and extension at 72°C for 30 s followed by a final extension at 72°C for 5 min. 5 microliter PCR product was analyzed by electrophoresis (Bio-Rad) in 1% Agarose (SIGMA) gel, at 100 volts for 40 min, followed by staining with 1% solution of ethidium bromide (50 microliter/L). Gels were visualized by UV transillumination.

### Characterization of probiotic properties

Acid tolerance was studied using acidic PBS (0.03 g KH_2_ PO_4^-^_, 0.18 g Na_2_ HPO_4_ 2H_2_O, 0.18 g NaCl for 20 ml PBS) and neutral PBS (18, 19). To do this, bacteria were grown in 50 ml of MRS broth at 37°C for 40 hours. After 2 days, this suspension was centrifuged at 3000 rpm for 15 minutes after the pellets which were resuspended in 20 ml acidic PBS (pH 2.5) and anaerobically and aerobically incubated at 37°C and then centrifuged at 5000 rpm for 30 min. The pellets were resuspended into the 20 ml neutral PBS and centrifuged at 3000 rpm for 15 minutes. Subsequently, the pellets were resuspended into few saline solution and 100 l from suspension was added into 9.9 ml saline solution. Serial dilution was performed until 10^−8^ dilution spread on MRS agar petridish. From 10^−6^, 10^−8^ dilutions, 100 μl suspension was spread on MRS agar plates. The plates were incubated anaerobically and aerobically at 37°C for 2 days. This work was perfomed for pHs 3.5, 4.5, 5.5, 6.5. The results were investigated (15, 16). For bile salts tolerance tests, the bacteria were cultivated into the MRS broth containing 0.3% deoxycholate sodium (bile salt). From cultivation time to later 8 hours. The absorbance was measured by the spectrophotometric method (OD_600_) every 30 minutes (15, 16). To distinguish heterofermentative or homofermentative nature of the isolates, the bacteria were cultured into a Gibson semisolid medium and incubated at 37°C anaerobically and aerobically. The Gibson medium contained: 2.5 g meat extract, 50 g Glucose, 100 ml tomato juice, 800 ml fat free milk,10 ml of % 0.4 MnSO_4_, nutrient agar (for 200 ml was added) and distilled water was added to make 1000 ml medium (15, 16).

### Microencapsulation

Sodium alginate, Xanthan gum, Chitosan from Sigma Aldrich (Canada) and Calcium chloride, Tween 20 (polyoxyethylene sorbtan monolaurate), Glycerol and KCl – HCl buffer solution of pH 2.0 from Merck (Germany) were purchased.

The microencapsulation procedure was performed according to the method of Lee JS *et al.* (2004) (17). The alginate mixture was prepared by adding % 2 (w/v) alginate, % 5.5 (w/v) MRS broth, % 5 (v/v) glycerol, % 0.26 (w/v) Xanthan gum, % 0.1 (v/v) Tween 20, and % 20 (v/v) cell suspension into distilled water and then mixed together. *Lactobacillus* and *Enterococcus* isolated from silage were used to inoculate MRS broth for 40 hours at 37°C anaerobically and aerobically. After 2 days, the cells were harvested by centrifugation at 1500 g for 15 minutes at 25°C and pellets were resuspended into the MRS broth and divided into two parts: One part was used for microencapsulation and the other was used as free cells without capsule (for control) (18, 19). This work was performed for *Lactobacillus plantarum* ATCC 8014 and *Enterococcus faecium* PTCC 1393 as control. The mixture was injected through a flask containing 1000 ml of 0.5 M CaCl_2_ solution with gentle stirring with a magnetic bar. The microparticles formed were allowed to harden in CaCl2 solution for 15 minutes after which and filtered alginate microparticles were rinsed twice with sterile distilled water and then transferred to a solution of chitosan. Chitosan (0.4 g) was dissolved in 90 ml distilled water acidified with 0.4 ml of glacial acetic acid to achieve a final concentration of 4 g/L (19). Subsequently, washed beads were immersed into 100 ml of chitosan solution and stirred gently with a magnetic bar for 15 minutes to coat the surface of the alginate microparticles. The resulting chitosan – coated alginate microparticles were again separated by paper filteration and stored in a sterile plates, and placed at 4°C, 8°C, 25 °C. Microparticles were obtained after 24 h and liquefied by aseptically adding 1 g of bead to 99 ml sterile %1 sodium citrate (Merck) solution (pH 6.0) and then plate count was performed. This work was performed for 1 week and 1 month later (18, 19).

### Preparation of silage

Maize forage was harvested at the milk stage chopped to about 1.5 cm, treated with inoculant, enzymes, inulin and ensiled in 10 plastic bags and 10 glass jars. Each jar was filled with approximately 340 g (wet weight) of chopped forage. The bags and jars were stored at ambient temperature (20±2.5 EC). At the end of the ensiling period (37 d), jars from each treatment were sampled for chemical and microbiological analyses on days 7, 14, 30 and 37 after ensiling. Change in pH, and numbers of yeast and molds serve as spoilage indicators. The following treatments were applied to fresh forages: 1) negative control (no additives); 2) a combination of isolated *Lactobacillus* and *Enterococcus* (1 × 10^7^ cfu/g) and *L. plantarum* and *Enterococcus faecium* (1 × 10^7^ cfu/ g)(coated bacteria); 3) a combination of isolated *Lactobacillus* and *Enterococcus* (1 × 10^7^ cfu/g) and *L. plantarum* (1 × 107 cfug) (fresh bacteria); 4) 8 g inulin in 340 g silage (2, 3); 5) a combination of inulin and fresh bacterial inoculants (20, 21); 6) cellulase and α - amylase complex added to 340 g of wet forage yielded a final application rate of 0.6 PFU/340 g (22–24); 7) positive control (fresh bacteria + inulin + enzymes) (22, 23).

### Analysis of probiotics, molds and yeasts growth in the silage

Chemical analysis was performed in triplicate and presented on DM basis. The DM content of the fresh materials and silages was determined by oven drying for 48 h at 60°C. Crude protein was determined by the Bradford assay. Microbiological analysis was performed on pooled samples of the three replicate silos per treatment, per time point, except for replicate samples, which differed considerably in their appearance. Microbiological evaluation included enumeration of lactobacilli on pour plate MRS agar and yeast and molds on spread-plate malt extract agar. The other data were analyzed as a completely randomized design and subjected to Excel. Differences among means were tested using Tukey's test (Minitab software) and significance was declared at P < 0.05 (5–23).

## RESULTS

### Morphological and biochemical properties

The isolated bacteria were gram-positive, catalase-negative and heterofermentative bacilli that have yellowish, mocoid, rounded colonies. The carbohydrates fermentation pattern of *Lactobacillus* and *Enterococcus* are shown (Tables 1 and 2).

**Table 1:** Pattern of carbohydrates fermentation in isolated *Lactobacillus*.

Specie	Glucose	Galactose	Lactose	Ribose	Sucrose	Manitol	Arabinose	Fructose
***Lactobacillus***	+	+	+	+	-	+	+	+

**Table 2:** Pattern of carbohydrates fermentation in isolated *Enterococcus*.

Specie	Glucose	sucrose	Galactose	surbose	Manitol	Ribose	Arabinose
***Enterococcus***	**+**	**+**	**+**	**-**	**-**	**+**	**+**

**Fig. 1:**
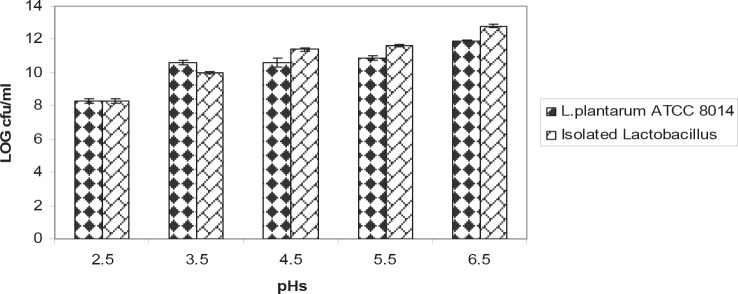
Comparison of acid tolerance between isolated *Lactobacillus* and standard strain *Lactobacilllus plantarum ATCC 8014* at 37°C. Results are indicated by the vertical bars. All mean survival rates were significantly different (p < 0.05).

**Fig. 2:**
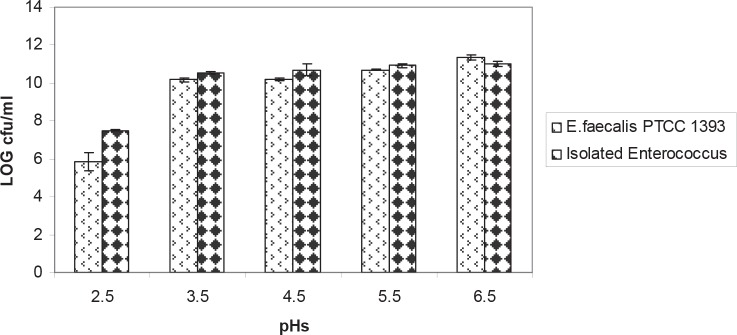
Comparison of acid tolerance between isolated *Enterococcus* and standard strain *E.faecalis* PTCC 1393 at 37°C. Results are indicated by the vertical bars. All mean survival rates were significantly different (p < 0.05).

**Fig. 3:**
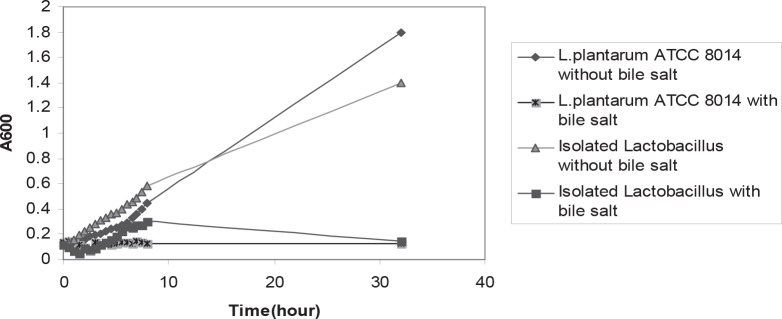
Comparison of bile salt tolerance between isolated *Lactobacillus*, standard strain *Lactobacillus plantarum* ATCC 8014 at 37°C. All mean survival rates were significantly different (p < 0.05).

**Fig. 4:**
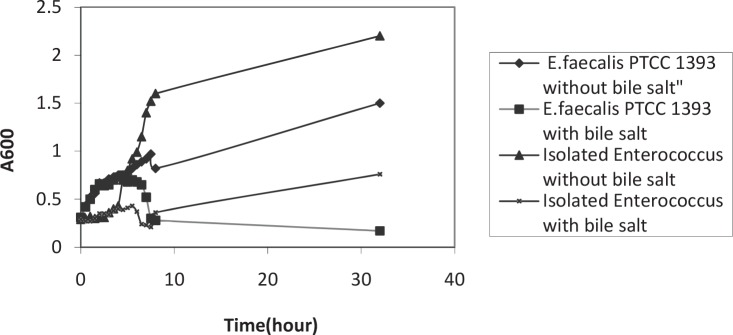
Comparison of bile salt tolerance between isolated *Enterococcus*, standard strain *E.faecalis* PTCC 1393 at 37°C. All mean survival rates were significantly different (p < 0.05).

The isolated bacteria were grown at 10°C, 50°C. These bacteria hydrolyzed arginine and produced NH_3_. The acid tolerance pattern and count of bacteria in different acidic pHs was shown in the Figs. 1 and 2. The results of bile salt tolerance were shown in the Figs. 3 and 4.

**Fig. 5:**
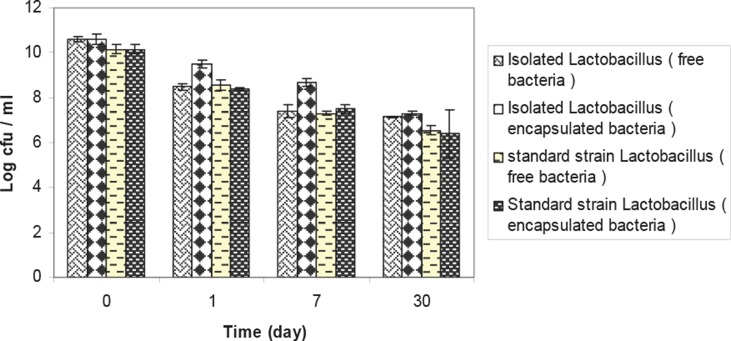
Comparison of viability between of fresh and encapsulated isolated *Lactobacillus*, standard strain *Lactobacillus plantarum ATCC* 8014 at 8°C. Results are indicated by the vertical bars. All mean survival rates were significantly different (p < 0.05).

### Molecular study of the selected isolates

Sequencing of 16S rDNA produced by polymerase chain reaction of bacterial DNA using universal primers revealed that superior Gram positive isolates were closely related to *Lactobacillus* spp., including *L. kefiri* and *Enterococcus faecium*.

### Morphological analysis of microcapsules

The size of microcapsules was 1-2 mm. They were stored at 4° C, 8° C and 25° C, were studied for morphologic alterations and size. The size of microcapsules (8° C, 25°C) was not altered after 1 month, but microcapsules that were stored at 4° C showed a reduction in size. After 1 month, microcapsules that were stored at 25° C were contaminated by fungi.

### Release of microparticles and study of cell viability

**Fig. 6:**
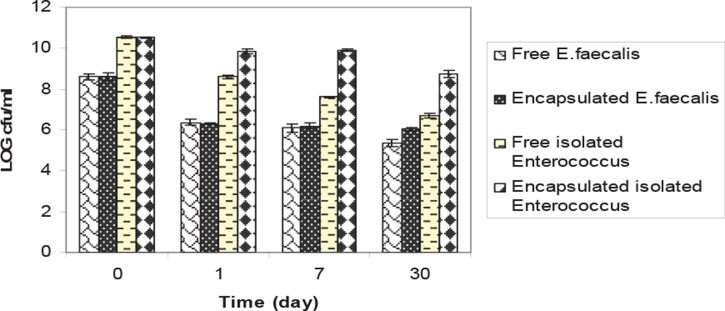
Comparison of viability between of fresh and encapsulated isolated *Enterococcus*, standard strain *E.faecalis* PTCC 1393 at 8°C. Results are indicated by the vertical bars. All mean survival rates were significantly different (p < 0.05).

After releasing of cells from microcapsules, enumeration of microorganisms by spectrophotometric and plate count methods was performed and the result is shown in Figs. 6 and 7. The viability of isolated *Lactobacillus* and *L. plantarum* in chitosan -coated alginate microparticles decreased from 3 × 10^11^ to 2 × 10^7^ cfu/mL. The viability of isolated *Enterococcus* and *Enterococcus faecium* in chitosan -coated alginate microparticles decreased from 3 × 10^11^ to 3 × 10^7^ cfu/mL.

### Analysis of probiotics, molds and yeasts growth in the silage

The chemical and microbiological compositions of the corn silage are given in Tables 3 and 4. Yeasts and molds had a low increase in any of the laboratory silages at any sampling time. After 37 days of ensiling there was a large increase in lactic acid bacteria (from 5log10 to > 11 log10 cfu/g), a decrease in pH and dry matter, and a low increase in molds and yeasts. Silage treated with *Lactobacillus plantarum* ATCC 8014 and *Enterococcus* faecium PTCC 1393 also had larger increase in lactic acid bacteria than did control silage.

**Table 3:** Chemical analyses of corn silage (means ± SD).

Agents/Silo	Day	1	2	3	4	5	6	7
	0	4.5	4.5	4.5	4.5	4.5	4.5	4.5
	7	4.6	4.5	4.4	4.4	4	4.3	4.3
**pH**	15	4.8	4.3	4.6	4.2	4	4.1	4.2
	30	5	4.2	4.2	4.2	4	4.2	4
	37	5.2	4.1	4	4.1	3.9	4.1	3.8

	0	3.48	3.48	3.48	3.48	3.48	3.48	3.48
	7	1.7	1.4	1.2	1.2	1.2	1.4	1.1
**(DM) Dry matter**	15	2	1.2	1.2	1.3	1.1	2.4	1.2
	30	1.3	1.39	1.3	1.3	0.92	1.3	1.2
	37	1.4	1.3	1.4	1.34	1	1.5	1.6

	0	91.57%	91.52%	91.52%	91.52%	91.52%	91.52%	91.52%
	7	91.57%	91.60%	91.51%	91.62%	91.54%	91.52%	91.66%
**% Crude protein**	15	91.52%	91.51%	91.58%	91.54%	91.53%	91.52%	91.52%
	30	91.53%	91.51%	91.66%	91.53%	91.52%	91.54%	91.52%
	37	91.52%	91.53%	91.60%	91.52%	91.53%	91.3%	91.53%

**Table 4:** Microbiological analyses of corn silage (means ± SD).

Agents/Silo	Day	1	2	3	4	5	6	7
	0	4.8	4.8	4.8	4.8	4.8	4.8	4.8
	7	5.2	7.3	5.6	7.5	7.1	5.3	6.1
**Molds & yeast** Log Cfu/ml	15	6.3	8.3	7.9	7.9	7.3	5.5	5.3
	30	8.6	7.08	7.8	7.2	7.2	7.6	6.6
	37	9.2	8.2	7.2	7.6	6.6	7.9	6.9

	0	5.3	5.3	5.3	5.3	5.3	5.3	5.3
**Probiotics** Log Cfu/ml	7	8.9	9.5	9.6	9.1	9.1	8.1	9.8
	15	10.6	9.6	10.4	9.2	10.5	9.4	11.5
	30	10.8	9.7	11.7	10.5	11.7	10.7	11.6
	37	11.3	10.1	12.3	11	12.2	11.3	12.4

## DISCUSSION

The mechanism of effectiveness of a probiotic is closely associated with the properties of the production of strains (25, 26). producing it is Lactobacillus are often found in association with plant materials, dairy products and as the dominant microbial population on forage crops and silages (27). Many studies (2, 3) have reported that the inoculation of forage with homofermentative *Lactobacilli* such as *L. casei, L. plantarum* have beneficial effects on promoting lactic acid fermentation and improving silage quality. However, the heterofermentative *Weissella* and *Leuconostocs* did not improve silage quality and may have caused some fermentation loss.

In this study, we isolated heterofermentative acid tolerant *Lactobacillus* spp. strains from corn silage. The isolated *Lactobacillus* was bile salt intolerant. The isolated bacterium showed significant tolerance to bile salts, however, when the tolerance level was compared with the standard strain, the difference was insignificant.

Microencapsulation techniques have been successfully used to enhance dairy fermentation for the production of concentrated lactic acid bacteria and to improve the survival of microorganisms in dairy products. Studies of Lee *et al.* (2004) have shown that both free and microencapsulated cells show similar stabilities during 4 weeks of storage at 4°C (17). Microencapsulation of the isolate with alginate and chitosan showed higher stability than free cell culture during a month period. The difference between encapsulated isolated *Lactobacillus* and standard strain *Lactobacillus* was less significant (p < 0.05). The results in the current study indicate clearly that inoculation with isolated *Lactobacillus* alone or in combination with a homofermentative LAB improved the aerobic stability of low-DM corn silage. Isolated *Lactobacillus* + *L. plantarum* inoculated silages had higher decreases in pH than control (P < 0.05). These findings are in agreement with corn silage findings (2–5). The results also show that the combination of L. *plantarum* with isolated bacteria is preferable because the combination accelerates the initial lactic acid fermentation rate, reducing pH and giving lower protein degradation and fermentation losses.

Addition of a microbial inoculant to corn silage resulted in greater production of acid early during ensiling, which caused a more rapid drop in silage pH. Addition of enzymes and inulin helped to better growth probiotics and more stability silage. Treatment of silage with cell-wall degrading enzymes did not consistently affect pH (22, 23, 28–30). These findings are in agreement with those of with corn silage. However, ensilage of these corn silages with bacterial inoculants, enzymes and inulin in laboratory-scale mini-silage demonstrated positive effects on silage fermentation and composition of this enzyme-inoculant mixture.
